# The Use of Big Data in Personalized Healthcare to Reduce Inventory Waste and Optimize Patient Treatment

**DOI:** 10.3390/jpm14040383

**Published:** 2024-04-03

**Authors:** Yara Badr, Lamis Abdul Kader, Abdulrahim Shamayleh

**Affiliations:** 1 Department of Biomedical Engineering, American University of Sharjah, Sharjah 26666, United Arab Emirates; g00093100@aus.edu (Y.B.); g00068616@aus.edu (L.A.K.); 2 Department of Industrial Engineering, American University of Sharjah, Sharjah 26666, United Arab Emirates

**Keywords:** precision medicine, big data, challenges, opportunities, benefits, stakeholders, solutions, applications

## Abstract

Precision medicine is emerging as an integral component in delivering care in the health system leading to better diagnosis and optimizing the treatment of patients. This growth is due to the new technologies in the data science field that have led to the ability to model complex diseases. Precision medicine is based on genomics and omics facilities that provide information about molecular proteins and biomarkers that could lead to discoveries for the treatment of patients suffering from various diseases. However, the main problems related to precision medicine are the ability to analyze, interpret, and integrate data. Hence, there is a lack of smooth transition from conventional to precision medicine. Therefore, this work reviews the limitations and discusses the benefits of overcoming them if big data tools are utilized and merged with precision medicine. The results from this review indicate that most of the literature focuses on the challenges rather than providing flexible solutions to adapt big data to precision medicine. As a result, this paper adds to the literature by proposing potential technical, educational, and infrastructural solutions in big data for a better transition to precision medicine.

## 1. Introduction

Healthcare practitioners oversee various data, including the patient’s medical history (diagnoses and prescriptions), medical and clinical data (imaging and laboratory procedures), and other private or confidential medical information. Previously, it was a common practice to keep patients’ medical records in handwritten notes or typed reports [1]. However, with the development of computer systems, clinical tests and records are increasingly digitized into electronic records known as Electronic Health Records (EHRs). EHRs are a digital version of medical records that contain information about a patient’s past, current, or future physical or mental health or condition [2]. Meanwhile, the concept of “big data in healthcare” is growing as a result of the integration of healthcare payer–provider data such as EHRs, pharmacy prescription, imaging, and insurance records along with genomics-driven experiments such as genotyping and gene expression data. In addition, recently, vast amounts of data are being collected in real-time from wearables, smart phones, smart devices, chips, and other data acquired through the smart web of the Internet of Things (IoT). The main goal of integrating big data in healthcare is to improve healthcare quality, service efficiency, and costs and reduce medical errors [3].

Precision medicine is a concept that can potentially transform medical interventions by providing effective, tailored therapeutic and treatment strategies based on an individual’s genomics and omics profiles. The 21st century vision of precision medicine is to provide ‘the right drug, with the right dose at the right time to the right patient’ [4]. It is currently a novel topic in the healthcare industry. Precision medicine involves tailoring a treatment specific to an individual with a disease. Furthermore, it helps in the prevention of disease. Increased utilization of molecular and genomic stratification of patients, for instance, assessing for mutations that give rise to resistance to certain treatments, will provide medical professionals with clear evidence upon which to base treatment strategies for individual patients. With this development, there will no longer be a dependence on the adverse outcomes of trial and error in prescribing methods and drug delivery.

Currently, when the prescribed medication is ineffective, the patient may be switched to a different medication. This trial-and-error approach leads to poorer outcomes for patients in terms of adverse side effects, drug interactions, and potential disease progression, meaning effective treatment is delayed and patients are dissatisfied [5]. Moreover, it also affects the healthcare industry in terms of wasted time, wasted inventory, and the overall quality of hospital performance. On the other hand, precision medicine is expensive due to the high cost of the needed technology.

In precision medicine, there are high expectations for genomics data to provide clues on what causes a disease’s initiation and progression and to enable the development of new strategies for disease prediction, prevention, and treatment. The idea is to translate omics profiles into subject-specific care based on their disease networks [6]. However, the ability to decipher molecules and their mechanisms remains limited, despite growing access to omics profiles, due to the complexity of the biological processes, limitations in statistical analysis, and cellular heterogeneity, thus, causing a bottleneck effect. Since then, the bottleneck has shifted from data generation to interpreting, analyzing, integrating, and managing the data. Therefore, informatics–computational technology is an essential component of precision medicine. Ultimately, integrating big health data into precision medicine could be costly at the beginning due to the investment in new technology. Still, it will be provided to later generations at a lower cost and more efficiency.

Integrating big data to develop precision medicine in healthcare seems to be a promising direction for improving patient treatment and care and the allocation of resources by the healthcare service, reducing wasteful expenditure and improving the overall quality of healthcare. However, although precision medicine is becoming the new trend, the healthcare sector face many challenges in integrating big data and precision medicine into their practice, which need to be discussed and considered. On the other hand, big data and precision medicine offer many benefits that are often underestimated or unnoticed. Therefore, it is important to identify the benefits that can be gained from such integration.

This work discusses the limitations of precision medicine to answer the following questions: Is the hindrance to utilizing precision medicine in healthcare due to a lack of knowledge in integrating new fields in ‘physician practice? On the other hand, what benefits does precision medicine bring forward that aid in recognizing its value? What are potential solutions that could fix the limitations and promote precision medicine? Furthermore, this work proposes solutions to integrate big data and precision medicine in the healthcare industry.

The remainder of the paper is organized as follows: The proposed methodology is described in Section 2. The results and the literature analysis are presented in Section 3. We discuss the implications of the study and present conclusions and directions for future research in Section 4.

## 2. Materials and Methods

Two research questions directed this literature review. The first question explores the challenges of implementing big data in precision medicine. The second question explores the benefits of big data being implemented with precision medicine. A systematic literature review was performed. The review structure follows the Preferred Reporting Items for Systematic Reviews and Meta-Analyses (PRISMA) guidelines.

### 2.1. Literature Search and Inclusion Criteria

The research papers used in this literature review were extracted from online databases, including peer-reviewed papers, conference records, and book chapters. In addition, some previous systematic review papers were also included to benefit from the assimilation of past knowledge. Finally, specific search filters were used as a practical scheme for retrieving related articles from various databases. The database sources that were used are IEEE Xplore Digital Library, PubMed, Science Direct, ProQuest, Elsevier, Research Gate, and Google Scholar.

The bibliography table was used to generate a list of the keywords that could be used in the literature search process to obtain the relevant articles. Boolean operators such as “AND” and “OR” were used to refine the search. The search variations were mainly in the form (ab(big data) OR ab(personalized)) AND (ab(challenges) OR ab(limitations)) AND (ab(benefits) OR ab(opportunities)). The retrieved papers were qualitative and theoretical. The most common report styles included comprehensive case studies of a sector and reviews of different limitations and challenges that prevent big data and precision medicine implementation in healthcare. Furthermore, it suggests some solutions and large advantages that can result from adding it as a standard to the healthcare industry. Some research formats included randomized controlled trials, surveys, case studies, and examples of applications that apply big data in healthcare.

### 2.2. Screening and Coding Procedures

The number of records initially identified via database searching was 556 studies. Next, the articles with related titles or relevant abstracts were inspected and held for further analysis. For inclusion, the selected papers are either scholarly journals or studies, and the content is related to the review questions. After excluding the duplicates, the articles were further screened to exclude articles that did not accentuate the topic of big data in precision medicine. As a result, 150 studies were identified from the used databases.

**Figure 1:**
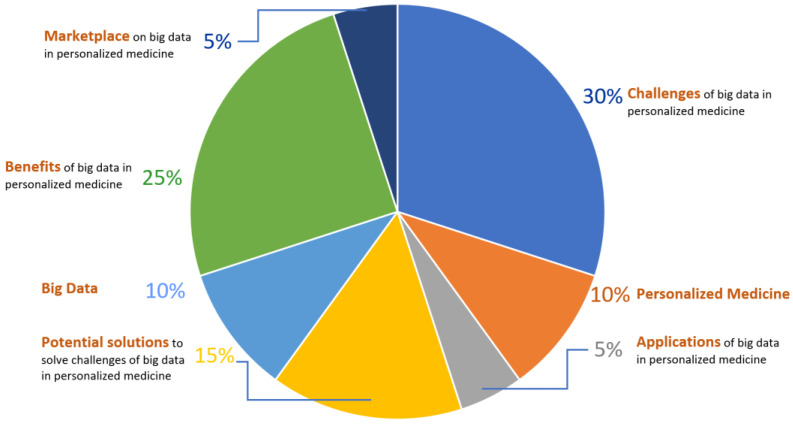
Analysis of the references used.

**Figure 2:**
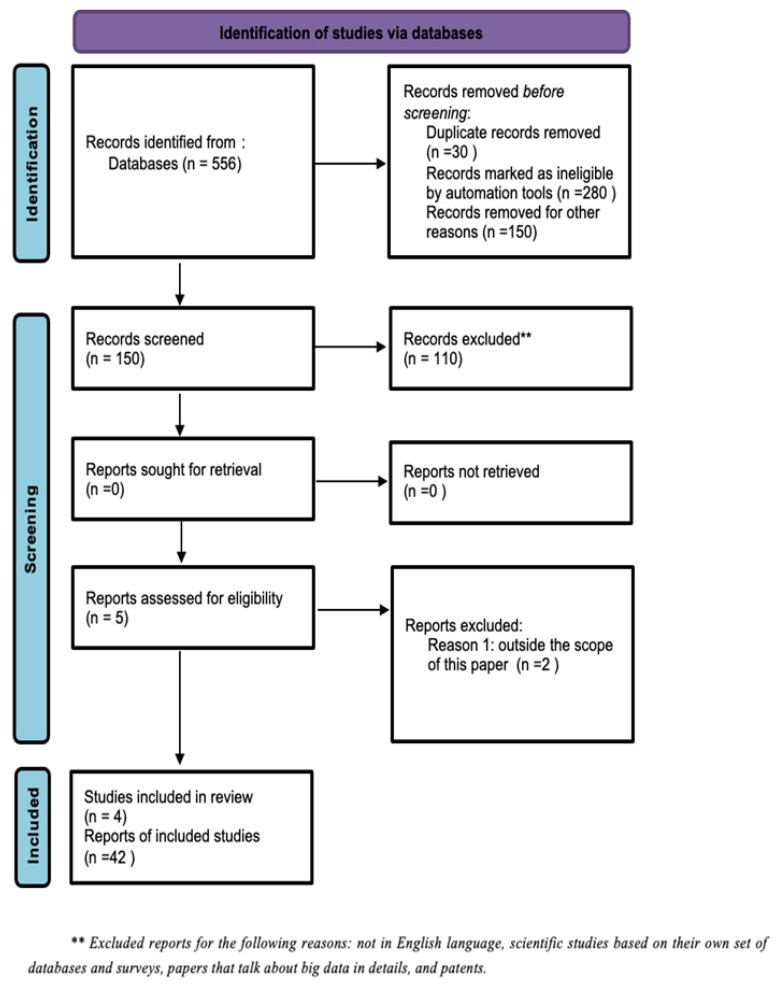
PRISMA diagram.

Additionally, the reference list of all the selected papers was searched. Finally, 46 articles were used for this literature review highlighting the trend of precision medicine using big data. Figure 1 shows an analysis of the percentage of references out of a total of 46 that convey each topic in this paper, while Figure 2 shows the PRISMA Flow Diagram. Finally, supplementary to the screening procedure, simple bibliographic data were tabulated for the genesis of ideas and we analyzed the research design.

## 3. Challenges Associated with Big Data and Precision Medicine

Clinical practice has been slow to incorporate precision medicine. Behind this lag in clinical adoption, there are several challenges that many healthcare delivery systems are facing as they attempt to adapt to the new requirements, practices, and standards.

In this review, challenges from different perspectives that were discussed in several studies were collected and are listed in detail in Table 1. These challenges have been classified into five main categories: awareness and education, patient privacy/data collection, value recognition, data management and infrastructure, and other issues.

### 3.1. Awareness and Education

One of the main challenges facing healthcare in incorporating precision medicine is the lack of awareness, which can occur from the healthcare sector or the patient side.

#### 3.1.1. From the Healthcare Sector Side

A survey claimed that only four out of ten consumers are aware of precision medicine. At the same time, only 11% of the patients reported that their doctor had discussed or recommended precision medicine treatment options [24]. Precision medicine is not a common topic discussed at the point of care. This can occur primarily because clinicians are not fully aware of this new area or are hesitant about implementing it in their practice, believing it is time-consuming and too burdensome [25]. This lack of discussion between clinicians and their patients has several adverse effects, including poor knowledge and awareness about precision medicine among patients, which consequently leads to low demand in that area (leading to limitation (Section 3.1.2)).

Moreover, integrating precision medicine into medical practice requires using new technology and understanding the technique. In addition, due to the heterogeneous nature of omics data and EHRs, hybrid knowledge about human genomics, diseases, and various analysis algorithms for integrating and interpreting these data are required. Unfortunately, this skill is not yet common among professionals, and there is a noticeable lack of training [21].

#### 3.1.2. From the Community/Patient Side

On the other hand, apart from the low demand, due to the lack of community education, the terminology “precision medicine” has been used interchangeably with other terms such as “personalized healthcare”, “stratified medicine”, “personalized medicine”, “individualized medicine”, and more, which has led to a misunderstanding about its benefits. However, several studies have tackled this issue by explaining how these terminologies differ [26]. Precision medicine focuses on tailoring treatments based on individual characteristics like genetics, environment, and lifestyle, aiming for the right treatment for the right patient at the right time. Personalized medicine shares this ethos but encompasses broader patient needs and preferences, emphasizing patient-centered care. Stratified medicine categorizes patients into subgroups based on shared characteristics to optimize treatment efficacy. Individualized medicine tailors interventions to the unique needs of each patient, considering genetics, environment, lifestyle, and personal preferences. In addition, there is a developing process of accommodating education about personalized medicine in the study curriculum, not only targeting medical faculties/universities and practitioners but also the wider public, potential patients, and real patients.

### 3.2. Patient Privacy/Data Collection

Data collection and patient privacy are interconnected in healthcare. The ownership and control of patient data, protection of sensitive information, etc., are all important considerations to ensure that data collection respects patient privacy rights. The collection of information to develop big data is the first step in adopting precision medicine. However, healthcare providers tend to face many challenges in data collection. First, there is a common misconception about data ownership, where patient data seem to belong to the institution; however, it is the property of the patient, and accessing and using it outside of the professional sphere necessitates patient consent [27]. Furthermore, due to the ownership misconception, health providers usually do not involve patients’ desires in their healthcare decision-making [25]. Meanwhile, to use the patient’s information, permission must be obtained in the form of signed consent.

Obtaining patient consent requires direct interaction of the patient with on-site staff [13,27], which is time-consuming and costly. Apart from that, patient protocols for the use of molecular data are frequently unclear or inappropriate, leading to misinformation about how the data are going to be used [25,28]. In addition to that, medical records, which include patients’ personal information, are usually tightly secured and not made public, which makes data collection challenging. In gathering non-medical data, usually, hypothesis-driven research is utilized, in which data are destroyed after being used [27]. However, in healthcare, such practice cannot be adopted as the precision medicine concept revolves around the use of big data (i.e., a large amount of accumulative data); thus, destroying the data would ruin the concept of precision medicine. Furthermore, apart from the information being confidential, data security is very challenging, as there are concerns regarding the insecurity of a patient’s molecular data and its vulnerability to attacks [25,29]. In comparison, the collection of non-medical big data is characterized by low cost, low information density, and is mainly gathered by chance. Clinical big data, on the other hand, are known to be of high information density. It needs to be acquired intentionally and under informed consent, making it difficult, costly, and time-consuming [27].

### 3.3. Value Recognition

Before implementing any plan or practice in an organization, ensuring the workforce is knowledgeable about it is important. They must also understand and recognize its value and the benefit it will bring. This will enhance their commitment to adopting it in their practice. However, that is not the case with precision medicine, as it is a new practice being implemented. There seems to be an exigent challenge in identifying the value of precision medicine, and the benefits of incorporating it into healthcare are not yet fully recognized [30]. This leads to another challenge, i.e., it is yet unclear how to convince physicians of the value of precision medicine so that they adopt this new technology into their clinical practice [7]. Many clinicians believe collecting, storing, and analyzing patients’ molecular information for precision medicine requires more time than they believe it is worth. On the other hand, some clinicians are hesitant to implement precision medicine methods for several reasons, including their belief that it demands time and are not well compensated. In addition, it is too burdensome to involve genetics experts/counselors in patient care [25].

### 3.4. Data Management and Infrastructure

In this area of data management and infrastructure, the challenges faced by different organizations can be subdivided into three categories, i.e., lack of standardization; the storage, transfer, and management of data; and data integration issues.

#### 3.4.1. Lack of Standardization

First, as stated before, precision medicine is a new practice that is being implemented in the medical field; as the healthcare (HC) setting has no standard protocols, this has led to ambiguity and challenges in adopting such practice. There is no consistent technique set to follow regarding the process of carrying out research and collecting clinical phenotypes, which clearly hampered the research from moving further. Furthermore, this inconsistency in carrying out the research has also led to the inability to achieve replicable clinical testing results [8]. Replication is one of the most important techniques for scientists to gain confidence in the scientific validity of their findings. When the findings of one study are consistent with another, it is more likely to be a trustworthy claim about the new practice [9]. Apart from that, Canada, for instance, lacks standardized quality assurance and regulated laboratory oversight. This means that there is an inconsistent technique for evaluating the clinical method adopted or approving the use of genetic testing in the first place. All of these factors result in the vagueness of the required standard of care [10]. Adding to that, even if the data were collected, there seems to be no standardized Electronic Health Record (EHR) that accommodates genetic data; if it did exist, it was not generalized or consistent among different healthcare services, research centers, and biobanks (within the same country or between different countries). Overall, due to the lack of consistency in the whole procedure from the technique of collecting data to the evaluation of the data and to storing it, there are no clear decision-making procedures in the existing precision medicine programs [25].

#### 3.4.2. Storage, Transfer, and Management of Data

Healthcare is now gathering new and diverse types of data, including healthcare provider data (such as EHRs, pharmacy prescriptions, and insurance records) and genomics data, to adopt the precision medicine concept. However, the amount of data collected continuously from patients in HC is significantly large and can be used to build the big data for precision medicine. However, there seems to be a limitation in compiling these data to form the big data required. The main cause is primarily because hospital medical data repositories were designed and built in the pre-big-data era to be standalone and siloed. Moreover, the current technological infrastructure of these standalone data repositories does not allow for the transfer, modification, and management of medical data. So, the velocity and amount of data required to adopt big data approaches are typically segregated in clinic or hospital charts, with no central sharing [27]. Moreover, current information technology systems are yet incapable of properly managing large volumes of patient molecular data. The current research laboratories, systems, and EHRs also lack the appropriate storage and computational resources required to integrate, manage, process, and analyze genetic data [11,25].

#### 3.4.3. Data Integration

Furthermore, the limitations of adopting precision medicine do not end at managing the data but also extend to how to integrate and make use of the large amount data that has been collected. Compared to big data from other fields (such as social media or advertisement), medical data are more complicated due to the combination of heterogeneous information. This means that, unless the data are processed and converted into an understood format, it is useless to the HC [27]. Unfortunately, over the last decade, a growing gap has become evident between researchers’ ability to extract and generate omics data and the ability to integrate and interpret these data [11]. Due to the previously discussed limitations (i.e., data collection and management), the major focus was on generating and storing the maximum possible amount of data, resulting in this gap. On the other hand, it was discovered that the process of extracting correlations to provide actual and meaningful biological interactions is not straightforward due to the computational difficulty of analyzing hundreds of variables (volume), which are also heterogenous in nature (variety) [11]. Overall, this leads to the inefficient use of individual molecular data in the provision of care [25].

### 3.5. Other Issues

Lastly, a controversial topic has been raised recently regarding the association of precision medicine with economic inequality and the generalized availability of treatment. It was claimed that adopting precision medicine in healthcare would further widen the economic inequality in the health systems between high- and low-income countries. Low- and middle-income nations may not be fully able to integrate such practices due to precision medicine’s financial and training requirements, which are not easily achieved in these nations. Additionally, the expenses of transitioning to precision medicine are still under study and not completely known [11]. Furthermore, even within high-income countries, precision medicine services are not always provided or available, especially in rural areas. Meanwhile, geneticists, genetic counselors, and molecular pathologists are also not always readily available in these areas. Consequently, many patients are either hesitant or unable to travel to other healthcare facilities to receive such services [7,25].

The challenges are categorized in Table 1.

## 4. Benefits of Big Data in Precision Medicine

### 4.1. Leveraging EHRs to Optimize Patient Health

In a healthcare setting, Electronic Health Record (EHR) data are structured and non-structured. The same thing goes for different industries in business. Thus, technology to deal with both will be required to allow straightforward interpretation. In business, this allows for identifying new opportunities and developing new strategies. Similarly, in a clinical setting, this will allow new interpretations, better understanding of the disease, and the development of new treatments [31]. Big data is the technology needed to change the typical clinical setting in healthcare today. Big data processes can collect and combine data from numerous sources like clinical tests, laboratory tests, imaging, genetics, and more in the clinical domain, thereby providing “intelligence observation” instead of observation derived from single-source data or the usual routine observation [32].

#### 4.1.1. Enhancing EHR Data Quality for Precision Medicine

EHRs with big data allow the study of virtually any disease and genetic influences that cause risk factors for a particular disease [33,34]. Furthermore, it will enable leveraging these data to study a spectrum of phenotypes for a particular disease, thereby giving rise to precision medicine. EHRs with big data have information that is preprocessed. Preprocessing involves the collection, standardization, and curation of data. Thus, data in EHRs under the concept of big data are standardized, more reliable, and accurate, thereby assuring the data quality of every patient’s EHR [33,34]. It is suggested that building EHRs with big data involves more than just integrating lab testing and genomic sequencing results. It also involves data mining for narrative text and recording lifestyle choices such as diet and exercise. In addition, more data like family history, prescribed drugs in the past and present, allergies, data from wearable technologies and imaging reports in unformatted free-form text, and discharge summaries exist in EHRs. Finally, the wealth of existing data that the EHR provides enables richer health and disease profiles to be studied at both individual and population levels.

EHRs will allow cost-effective and efficient implementation through smooth data exchange between the patient and the provider. We can consider the integration of pharmacogenomics into patient care as a primary example of the power of EHRs, whereby the healthcare industry is introduced to precision medicine [35,36].

#### 4.1.2. Integrating Precision Medicine in EHRs

A central data warehouse would provide a site for integrating various data, thus allowing curation, combination, and analysis of all data. Unfortunately, such centralized repositories still do not exist in Health Information Technology (HIT) and infrastructure within hospital settings. These data repositories were built and designed before the era of big data [14,15]. Hence, they are standalone and isolated, with no intention of allowing the data to be collected, combined, and then analyzed in conjunction with various data sets. Ultimately, this paper previews the need for newly installed information technology systems within clinical domains to ensure there are means to share data between systems, thus optimizing patient treatment [37].

#### 4.1.3. Empowering Patients in HER Management

When we look at patient data of any sort and format, because it is held within medical organizations it appears that it belongs to that organization. Nevertheless, these organizations merely act as custodians for that data, which should be the patient’s property alone [16,17]. Moreover, accessing and using data from outside the clinical realm will require the patient’s consent. Hence, this puts a brake on exploiting the large volume of data in clinical records [19].

In addition, when studies or cases are concluded, the data are destroyed, thus preventing the whole idea of using such cases for research and education. If big data technology is used, millions of data are processed and they are stored collectively in a patient’s profile. The idea of losing those valuable data when a case has ended is counter-intuitive to the advancement of medical knowledge and the concept of big data itself, as a result [20,22]. Patient consent to store and use the data is more powerful and allows the accumulation of large data sets, thus giving access to develop driven research questions and hypotheses base on those data.

### 4.2. Disease Prevention, Differential Diagnosis, and Disease Treatment

As mentioned earlier, individualized treatment/tailored pharmacotherapy is one of the important benefits of precision medicine implemented using big data. It also has more benefits:

- Disease prevention and prediction of disease risk before the symptoms belonging to that disease start to manifest [22].
- Differential diagnosis, involving the timely and instant identification of an illness [10,25].
- Disease treatment, including different strategies to develop the best-optimized treatment once the disease is identified.
- These benefits highlight the move from focusing on treatment only in healthcare to a system that includes prevention, prognosis, and post-disease survivorship as important aspects in healthcare, thus revolutionizing this industry [22].

#### 4.2.1. Disease Prevention

In the context of disease prevention, precision medicine aims to accurately predict the disease years in advance through genomics. Time and early detection are important factors for successful intervention to decrease the risks of many severe-health-outcome diseases, such as cancer. For these kinds of diseases, the etiology (cause of the disease) is not entirely understood. Thus, prediction modeling should be used to identify disease markers as early as possible. Such models can help change the outcome of the disease and give enough time for an intervention. Precision medicine through big data will help healthcare organizations to shift the emphasis in medicine from reaction to prevention [22].

#### 4.2.2. Differential Diagnosis

With big data in precision medicine, the timeframe for identifying and diagnosing a disease is reduced to days and can even be achieved in hours [34,38]. For example, acute abdominal pain can have a very different etiology, such as in kidney stones, aortic aneurysms, and more diseases. If the diagnosis is mistaken for a chronic condition, this can severely affect the quality of life. Nevertheless, this can be prevented by using genetic markers and metagenomics sequencing to screen for many diseases. Thus, big data through precision medicine will introduce the notion of “precise phenotyping” into the medical world and minimize trial-and-error inefficiencies that can inflate the health costs for patient care and treatment [17].

#### 4.2.3. Disease Treatment

Disease treatment is the last step after disease prevention and differential diagnosis. Using prediction models that can be easily created using big data in precision medicine, different outcomes for treatments can be obtained. The prediction model would look specifically at the patient’s status, which includes their genes, metabolic profile, and drug exposure. Also, it would look at various treatments such as new drugs, diet, physical activity, therapy, surgery, and more, thereby identifying the treatment that is most suitable for the patient’s healthcare and lifestyle conditions. Optimization of the treatment is seen as a big research problem and big data in precision medicine provides the means to be able to provide a hypothesis and methodology for this research question.

### 4.3. Precision Medicine Introduces Precision Public Health

The director of the Office of Public Health Genomics at the Centers for Diseases Control and Prevention (CDC) defined ‘precision’ in the context of public health as “improving the ability to prevent disease, promote health, and reduce health disparities in populations by (1) applying emerging methods and technologies for measuring disease, pathogens, exposures, behaviors, and susceptibility in populations; (2) developing policies and targeted implementation programs to improve health” [39]. The CDC’s priority goals are to detect early outbreaks of a certain disease in the community, modernize surveillance, and apply targeted health interventions. To achieve their goals, comprehensive real-time data are needed to learn about the health status of the public. In other words, they need a large variety of accessible data and sequencing genomics on a population level [36,40]. Ultimately, big data in precision medicine is key, emphasizing public data sharing to achieve precision public health and speed–accuracy–equity decisions made by the government for the sake of the public [39].

### 4.4. Reduction in Inventory and Waste in Costs

The emerging field of big data and precision medicine has aimed to enhance patient treatment and diagnosis using pharmaceutical products curated and customized to individual needs and diagnosis [38]. Thus, through precision medicine, we will eventually have less wastage of ineffective and costly therapies that patients must undergo to receive treatment. Furthermore, prediction modelling and forecasting can be used to determine the patient’s exact diagnosis and optimum treatment, allowing healthcare providers to order only the required and curated medicines [40]. As a result, healthcare institutions are likely to change their way of dealing with pharmaceutical companies. They are also unlikely to order general medicines in bulk, which the hospital might not otherwise use effectively, leading to the drugs becoming expired and the hospital suffering waste in terms of cost and inventory [21]. In return, the hospital is likely to order specific drugs that provide the best treatment based on that patient’s big data and genomics profile. It should be noted that big data does not only allow us to look at the genomics and overall tests of the patient [41]. It also allows us to look at the patient’s environmental, behavioral, and lifestyle aspects, thus enabling the selection of the medication that best suits them. This would reduce inventory waste massively in hospital settings [42].

### 4.5. Research and New Partnerships Due to Precision Medicine

The stakeholders in healthcare institutions usually revolve around the government, officials that work in the hospital, doctors, administrators, patients, and those who are inside the organization. However, with the help of precision medicine, new partnerships between scientists from a wide range of specialties can take place, thus promoting research and education, since better integration of EHRs in patient care will allow researchers access to medical data more easily [14,43,44,45]. Also, people from the patient advocate community, universities, pharmaceutical companies, and others can all be partners in the new healthcare institutions that revolve around big data and precision medicine. Ultimately, opportunities for millions of people to contribute to scientific research advancement and the whole community will be available [25]. Table 2 categorizes the benefits.

## 5. Discussion and Recommendations

Healthcare organizations face many challenges every day, as previously discussed in this paper. Moreover, other new challenges are being discovered that are hindering the efforts of these organizations and services in implementing precision medicine in their practice. However, precision medicine provides many opportunities and benefits that have not yet been wholly discovered and deserve to be tackled.

For that reason, this chapter is mainly oriented towards discussing and suggesting a few solutions that might help target the challenges previously discussed and, therefore, help to improve the implementation of precision medicine. The challenges were categorized into five main categories: awareness and education, patient privacy/data collection, value recognition, data management and infrastructure, and other issues.

### 5.1. Solution for Awareness and Education

Regarding the issue of awareness and education, there are two areas to be targeted: healthcare/medical workforce awareness and patient awareness. For medical practitioners, who are already in practice, the best way to raise awareness is through training and educational programs. The organization should encourage and provide this training to all of their workforce. Meanwhile, for medical students, the education curriculum must be updated in a way that not only familiarizes the students with the precision medicine concept but also integrates the concept of hybrid learning. Where able, medical and pharmaceutical colleges should provide major courses (apart from biological/medical courses) about how to integrate and interpret medical data (mainly omics data, which is heterogenous nature) using different methods of data analysis algorithms and compactional platforms.

Furthermore, healthcare professionals should also be taught some programming skills to be able to investigate better ways of analyzing omics data. Meanwhile, when healthcare organizations’ and services’ knowledge about precision medicine improves, this will help spread awareness to the patients and population. This can be achieved by carrying out campaigns (that will spread awareness about the topic), conducting clinician-to-patient talks during checkups, or by developing websites or pages within the hospital’s website that are made available to the public, which provide an overview of precision medicine and its benefits.

### 5.2. Solution for Patient Privacy

On the other hand, regarding the issue of patient privacy/data collection, it was stated that healthcare providers are facing many challenges in the process of data collection as well as problems in obtaining patients’ informed consent due to unclear protocols and misinforming patients about the purpose of informed consent. However, if, in an appropriate and clear way, the patients were to be fully informed about how their information would be used and the efforts that would be made to protect their genomics information, this should motivate the patients to provide their consent. Moreover, this will also provide the patients with knowledge regarding the options available for treatment. Thus, healthcare providers should be more likely to consider patients’ preferences in terms of treatment and prevention methods.

### 5.3. Solution for Value Recognition

Although stakeholders understand the benefit of personalized healthcare, they are still reluctant to use it due to a lack of evidence and supporting data on its overall success in healthcare institutions. Moreover, they are still reluctant to change policies and practices without evidential data that demonstrate economical and clinical value. The development of evidence to provide to stakeholders should be an important issue that scientists and companies that develop sophisticated technology for precision medicine pay attention to. Thus, awareness of this matter should be demonstrated before providing evidence for the profit of precision medicine [10,20,29,38]. Although data on patient survival, disease progression information, and risk reduction have begun to emerge, they are not reported properly enough to be shared with other stakeholders. Thus, it seems like there is a lack of evidence on the benefits of personalized healthcare. One way to start the process of sharing evidence is to develop policies that would protect patients’ confidential information while also developing a learning health system that provides a universally accepted and user-friendly way to systematically collect and share treatment or outcome data. Thus, implementing a learning healthcare system would produce effective information management and systems to easily share clinical data for all patients and analyze the patterns and profits produced by precision medicine [20].

### 5.4. Solutions for Data Management and Infrastructure

Due to the rise of big data in precision medicine, there is an increased need to store the information and data generated by institutions that are conducting big projects. Thus, computational solutions, for instance, cloud-based computing, have emerged. Cloud computing is the best storage model that can provide the elastic scale needed for DNA sequencing, whose rate of technological advancement is also increasing rapidly [35,43]. The security and privacy of personal medical and scientific data remain a challenge, regardless of the cloud solution used.

Solutions to deal with big data, especially when analyzing complex genomics information, include the use of graphics processing units (GPUs), which have the potential to improve the computational power compared to conventional processors even in cloud solutions. Compared with the currently used central processing units (CPUs), GPUs are highly parallel hardware providing massive computation resources. GPUs have been recently used for proteomic analysis and metagenomic sequence classification.

## 6. Conclusions

Although precision medicine is gaining recognition in the present day, there is still not a complete system that is fully based on big data in personalized healthcare due to a lack of evidence among stakeholders. However, when conducting this research and writing the review paper, we noticed that precision medicine is initiating many start-up and experienced companies to build systems and software tools that will become very important in the management of big data [35,43]. The tools initiated by these companies are used to integrate genomics and complex information into EHRs, collect complex information, visualize data, and interpret them. We can see that there are small, forward steps being taken towards precision medicine. With the solutions mentioned in this paper and sharing of evidence for the sake of developing a learning healthcare system, precision medicine will be pushed forward towards this next generation.

## Figures and Tables

**Table 1:** Challenges for big data in precision medicine.

Challenges Categories	Challenges Mentioned	References
3.1. Awareness and Education	3.1.1. From the Healthcare Sector Side: - Precision medicine requires hybrid knowledge due to the heterogeneous nature of omics data. - There is a lack of formal training, awareness, and understanding of precision medicine technology among many physicians. This raises concerns regarding their ability to meet the standard of care. 3.1.2. From the Community/Patient Side: - Precision medicine’s demand is low due to a lack of education. - The variety of omics terminologies might lead to misunderstandings among doctors and patients.	[7,8,9,10,11,12]
3.2. Patient Privacy/Data Collection	Patient Privacy: - Medical records, which include sensitive personal information, are tightly secured and not made public. - Patient data seem to belong to the institution; however, it is the patient’s property, and accessing and using it outside the professional sphere necessitates patient consent. - Data security is challenging as molecular data are frequently insecure and vulnerable to attack. - The patient’s desire for treatment/prevention methods is not always considered. - When it comes to genomics, patients and their families are frequently misinformed. Data Collection: - Non-medical big data are mainly gathered by chance, at no cost, and with low information density. In contrast, big clinical data are acquired intentionally, at great cost, and with high information density. - Hypothesis-driven research applied to non-medical data cannot be employed in healthcare. (Information is important and cannot be destroyed; there is a need to accumulate.) - Patient consent protocols for the use of molecular data are frequently unclear or inappropriate.	[7,13,14,15,16,17,18]
3.3. Value Recognition	- It is unclear how to convince physicians of the value of precision medicine and to adopt the new technology into their clinical practice. - The value of incorporating precision medicine within healthcare institutions is not recognized. - Collecting, storing, and analyzing molecular data takes longer than many clinicians believe it is worth. - Some clinicians are hesitant to implement precision medicine approaches since they demand time and it burdensome to involve genetics experts/counselors in patient care.	[7,19,20]
3.4. Data Management and Infrastructure	3.4.1. Lack of Standardization: - Clinical research is hampered by the lack of a consistent technique for collecting clinical phenotypes. - There is a lack of standardized quality assurance and regulated laboratory oversight for evaluating and approving genetic testing for usage and determining the standard of care. - Electronic Health Record data are not based on standards. 3.4.2. Storage, Transfer, and Management of Data: - The velocity and amount of data necessary for big data approaches are typically segregated in clinic or hospital charts, with no central sharing. - Medical data repositories were designed and built in the pre-big-data era to be standalone and siloed. Thus, centralized repositories are currently uncommon in hospital clinical information technology architectures. - Information technology platforms and systems are incapable of adequately managing large volumes of individual molecular data. 3.4.3. Data Integration Issues: - Medical data are more complicated and less “usable” than data delivered to large corporations, requiring processing to convert it into a format that can be used. - In the last ten years, a growing gap has occurred between the ability to generate omics data and the ability to integrate it. - The extraction of correlations as actual and meaningful biological interactions is not straightforward due to the computational difficulty of analyzing hundreds of variables.	[7,8,13,21,22,23]
3.5. Other Issues	- There is a lack of public funding at the provincial level for specific genetic tests linked to therapeutics. - Precision medicine may further widen the economic inequality in health systems between high- and low-income countries. Many low- and middle-income nations may lose out on precision medicine. - Geneticists, genetic counselors, and molecular pathologists are not always available, particularly in rural areas.	[7,8,23]

**Table 2:** Benefits of big data in precision medicine.

Benefit Category	Benefits Subcategories and a Brief Description	References
4.1. Leveraging EHRs to Optimize Patient Health	4.1.1. Enhancing HER Data Quality for Precision Medicine - Big data and precision medicine propose access to large omics (genomics, transcriptomics, proteomics, epigenomic, metagenomic, metabolomics, and neutronics) and enable modelling of complex biology interactions using integrated information from big data in EHRs. - Big data in precision medicine allows hospital institutions to increase their understanding of how drugs work, considering adherence, co-morbidities, interactions, and side effects. 4.1.2. Integrating Precision Medicine in EHRs - Big data in precision medicine provides prediction and forecasting tools for intelligence observation. - Big data provides an increased capacity for information systems to collect, store, and integrate large volumes of data. 4.1.3. Empowering Patients in HER Management - Patient consent empowers large data set accumulation for research and hypothesis development.	[10,13,19,20,22,29,33,34,35,36,38,43,46]
4.2. Disease Prevention, Differential Diagnosis, and Disease Treatment	4.2.1. Disease Prevention - Precision medicine predicts diseases in advance through genomics, allowing proactive intervention. - Early detection facilitates timely intervention, reducing risks for severe health outcomes like cancer. - Big data is precision medicine shifts healthcare emphasis from reaction to prevention, transforming the industry. 4.2.2. Differential Diagnosis - Big data in precision medicine accelerates disease identification to days or even hours. - Genetic markers and metagenomic sequencing prevent misdiagnoses in conditions like acute abdominal pain. - It introduces “precise phenotyping”, minimizing trial-and-error inefficiencies and reducing health costs. 4.2.3. Disease Treatment - Prediction models, facilitated by big data, tailor treatment outcomes based on patient-specific factors. - It enables the utilization of genes, metabolic profile, drug exposure, and various treatments for optimization. - It addresses a significant research problem, offering hypotheses and methodologies for personalized treatment.	[10,22,27,33,34,36,38,40,43]
4.3. Precision Medicine Introduces Public Precision Health	- Precision medicine enhances the ability to prevent diseases, promote health, and reduce health disparities in populations. - It enabled the application of emerging methods and technologies for measuring diseases, pathogens, exposures, behaviors, and susceptibility in populations. - It allows the development of policies and targeted implementation programs to improve public health. - Priority goals include the early detection of outbreaks, modernized surveillance, and the application of targeted health interventions. - Comprehensive real-time data and genomics sequencing at a population level are crucial for achieving these goals. - Big data in precision medicine facilitates public data sharing, leading to precision public health. - It enables government decisions for the public’s benefit in terms of speed, accuracy, and equity.	[22,36,39,40]
4.4. Reduction in Inventory Waste and Costs	- Precision medicine customizes pharmaceutical products based on individual needs, reducing ineffective and costly therapies. - Prediction modeling and forecasting determine accurate diagnoses and optimum treatments, minimizing unnecessary medicine orders. - It may shift healthcare institutions from bulk ordering general medicines to ordering specific drugs tailored to patient profiles. - It would eliminate wastage from expired drugs, improving cost efficiency and inventory management. - Big data considers not only genomics but also environmental, behavioral, and lifestyle aspects, ensuring the best medication in provided to patients.	[13,20,21,25,33,40,41]
4.5. Research and New Partnerships Due to Precision Medicine	- Precision medicine fosters new partnerships among scientists from various specialties, expanding research opportunities. - It enables better integration of Electronic Health Records (EHRs) in patient care and enhances researchers’ access to medical data. - It facilitates the involvement of people from patient-advocate communities, universities, and pharmaceutical companies in healthcare institutions. - It creates opportunities for millions of individuals to contribute to scientific research advancements. - It strengthens community engagement and collaboration in the field of big data and precision medicine.	[14,25,43]

## Data Availability

Not applicable.
